# 3D localization of vena contracta using Doppler ICE imaging in tricuspid valve interventions

**DOI:** 10.1007/s11548-022-02660-w

**Published:** 2022-05-19

**Authors:** Hareem Nisar, Djalal Fakim, Daniel Bainbridge, Elvis C. S. Chen, Terry Peters

**Affiliations:** 1 Robarts Research Institute, 1151 Richmond St., London, ON N6A5B7 Canada; 2 School of Biomedical Engineering, Western University, 1151 Richmond St, London, ON N6A3K7 Canada; 3 Schulic School of Medicine and Dentistry, Western University, 1151 Richmond St., London, ON N6A3K7 Canada; 4 Department of Anesthesia and Perioperative Medicine, London Health Sciences Centre, 339 Windermere Rd., London, ON N6A5A5 Canada; 5 Department of Medical Biophysics, Western University, 1151 Richmond St., London, ON N6A3K7 Canada

**Keywords:** Tricuspid valve, Cardiac interventions, Intracardiac echocardiography (ICE), Image guided systems, Vena contracta localization

## Abstract

**Purpose:**

Tricuspid valve (TV) interventions face the challenge of imaging the anatomy and tools because of the ‘TEE-unfriendly’ nature of the TV. In edge-to-edge TV repair, a core step is to position the clip perpendicular to the coaptation gap. In this study, we provide a semi-automated method to localize the VC from Doppler intracardiac echo (ICE) imaging in a tracked 3D space, thus providing a pre-mapped location of the coaptation gap to assist device positioning.

**Methods:**

A magnetically tracked ICE probe with Doppler imaging capabilities is employed in this study for imaging three patient-specific TVs placed in a pulsatile heart phantom. For each of the valves, the ICE probe is positioned to image the maximum regurgitant flow for five cardiac cycles. An algorithm then extracts the regurgitation imaging and computes the exact location of the vena contracta on the image.

**Results:**

Across the three pathological, patient-specific valves, the average distance error between the detected VC and the ground truth model is $({1.22 \pm 2.00})$mm. For each of the valves, one case represented the outlier where the algorithm misidentified the vena contracta to be near the annulus. In such cases, it is recommended to retake the five-second imaging data.

**Conclusion:**

This study presented a method for ultrasound-based localization of vena contracta in 3D space. Mapping such anatomical landmarks has the potential to assist with device positioning and to simplify tricuspid valve interventions by providing more contextual information to the interventionalists, thus enhancing their spatial awareness. Additionally, ICE can be used to provide live US and Doppler imaging of the complex TV anatomy throughout the procedure.

## Introduction

Previously labeled as the forgotten valve, the tricuspid valve (TV) and its repair surgeries have gained prominence recently [1–3]. For the longest time, it was believed that “the TV is designed to be(come) incompetent” [4] and that the valve will heal itself after a left-sided surgery is performed [5]. Research and experience have shown otherwise. When left untreated, even after mitral valve surgery, the TV can develop high-grade regurgitation disease [6, 7]. Tricuspid valve regurgitation (TR) is the most common valvular disease in the right side of the heart, characterized by the backflow of blood from the right ventricle to the right atrium, and can be organic or functional in nature.

Currently, there are several devices and procedures approved for tricuspid valve repair. These repair techniques can be classified into two major categories—annuloplasty and coaptation devices. The edge-to-edge repair techniques are particularly successful in treating severe TR with the benefits of reducing the need for hospitalization as a result of heart failure [8].

Earlier success in TV repair includes the use of the MitraClip on the TV to reduce the regurgitation by at least one grade [9, 10]. Since then, a specialized tool called TriClip (Abbott Vascular, Santa Clara, California) was developed as a safe and effective device for TV repair via the TRILUMINATE trial [11].

The leaflet repair via the TriClip is performed percutaneously via transfemoral access, and while a transjugular approach has also been developed, the transfemoral approach has shown superior performance [12]. The clip is deployed either using the triple-orifice technique or more commonly, using a bicuspidization method. In this latter technique, the clip is placed between the anterior and septal leaflets of the TV to achieve the best post-procedural outcomes [13]. Currently, this procedure is performed under general anesthesia and the combined fluoroscopic and transesophageal echocardiographic (TEE) imaging. The tools are inserted into the right atrium and maneuvered carefully and iteratively using control knobs, fasteners and levers to reach the TV in the right ventricle under image guidance [12]. Lebehn et al. [14] describe a protocol for TEE imaging during these various steps involved in the device positioning, where device positioning involves localizing the leaflet coaptation gap at the leaflet tips and the assessment of the regurgitation based on the vena contracta. This is followed by the positioning of clip arms perpendicular to the coaptation gap—one of the most critical steps during a TV repair intervention. Device positioning using these steps is an established procedure for left-sided interventions; however, the same task becomes much more meticulous for right-sided cardiac interventions due to the constraint nature of the TEE.

The tricuspid valve and its interventions have been declared “TEE-unfriendly”. The TV is located anterior to the mitral valve, rendering it challenging to image using a TEE probe [15]. The large distance between the TEE probe and the TV, combined with the non-perpendicular alignment of the sub-valvular apparatus also makes the TEE imaging of the TV more demanding [16]. Quite often, the acquired TEE images of the TV are of suboptimal quality due to the presence of shadowing and complex TV anatomy. In such cases, it is recommended to introduce intracardiac echocardiography (ICE) into the procedural imaging [12]. ICE imaging can not only aid in the imaging of leaflets and tricuspid annulus but also guide the deployment of the tool correctly.

ICE ultrasound provides high-resolution imaging of cardiac structures. Unlike TEE, the insertion of an ICE probe can be performed under local anesthesia only and without the need for specialized operator. ICE is also well tolerated by the patients. The major drawback of this technology is the high cost of each single-use probe, but it has the potential to offer a better cost/benefit ratio, by reducing the procedure times and length of post-op hospitalization in patients. ICE has made its mark in interventional cardiology for structural heart diseases and electrophysiology [17–19]. ICE has also been a favorable choice for the interventional imaging of the tricuspid valve, where it may be utilized for discerning the annulus from the leaflets and for guiding tool positioning and orientation [20]. In several studies, ICE is used in conjunction with fluoroscopy or TEE to guide the tools and repair the TV in both annuloplasty and edge-to-edge repair [21–25].

Image-guided systems (IGS) have helped simplify many interventions, as well as having made them safer and more reproducible [26]. In a meta-analysis comparing the efficacy of image-guided and standard cardiac resynchronization therapy in patients with heart failure, Jin et al. [27] demonstrated that a strategy of echocardiographic guidance was associated with improved outcomes compared with a routine strategy. IGS can greatly benefit TAVI procedure in patients with complex and unusual anatomy, such as bicuspid aortic stenosis and situs inversus totalis [28]. As the push toward less-invasive cardiac therapies continues, image-guided intracardiac visualization has received clinical exposure, as it has the potential to improve the precision and outcome of surgical procedures [26].

To facilitate the positioning of a TriClip device, the identification and localization of coaptation gap is a crucial step that can potentially be simplified by pre-mapping the 3D location of the coaptation gap prior to the device being positioned. This mapped location serves as an important landmark during the TriClip positioning stage. In ultrasound imaging, the neck of the regurgitant jet, as seen in the color Doppler, is called the vena contracta (VC) and it corresponds to the location of the coaptation gap. In this study we aim to map the coaptation gap by localizing the vena contracta in Doppler ICE imaging.

While there is currently no commercially available automatic VC and annulus detection system, several automatic VC quantification techniques have been published in the past for the assessment of mitral regurgitation using TEE [29] and TTE [30]. Sotaquira et al. [31] have developed an algorithm to automatically detect and quantify the shape of the effective regurgitant orifice area using 3D TEE, and Li et al. [32] have developed a rapid MVA tracking algorithm for use in the guidance of off-pump beating heart transapical mitral valve repair using 2D biplane TEE images. The eventual goal of these developments is the creation of an image-guided system (IGS) for cardiac interventions in order to provide more timely and accurate information to the interventionalists.

In order to assist the positioning of coaptation device at the site of regurgitation, we propose to use a tracked ICE probe with Doppler imaging to identify vena contracta from ultrasound images and representing its location in 3D space. Tracked devices can then navigate to reach the targeted vena contracta. To the best of our knowledge, this paper presents the first image-guidance system for tricuspid valve interventions. A proof of concept study, performed on a simple silicone wall phantom, has been conducted by our lab and accepted for publication at the SPIE 2022 conference. In this paper, we present a guidance system which uses ICE imaging and EM tracking technology to identify the site of regurgitation from a patient-specific tricuspid valve in a beating heart phantom. This system is developed on 3D Slicer and implemented as an open-source, one-click, 3D Slicer module. The module, as well as some test data along with a video demonstration, can be found at https://github.com/hareem-nisar/VC-localization.

## Materials and methods

### Materials

In this study, we used a 10-French, forward-looking and radial ${\hbox {Foresight}}^{\mathrm{TM}}$ ICE probe along with the Hummingbird Console (Conavi Medical Inc., Toronto, Canada) to acquire ultrasound imaging of the valve. The ${\hbox {Foresight}}^{\mathrm{TM}}$ ICE is unique as it provides radial ultrasound as well as Doppler imaging capabilities [33], thus enabling the direct visualization of the anatomy and the regurgitation during interventions.

**Fig. 1:**
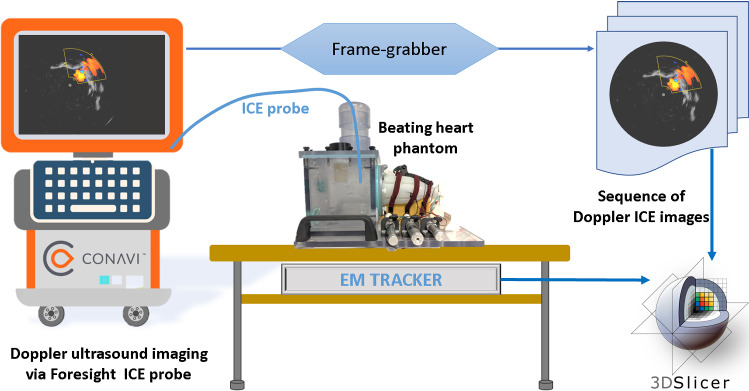
Experimental setup—ultrasound images are acquired using a frame grabber from the Conavi’s Hummingbird console. A tracked ICE probe is positioned inside a beating heart phantom to image the patient-specific tricuspid valve. Image and tracking information is sent to a 3D Slicer module for processing

**Fig. 2:**
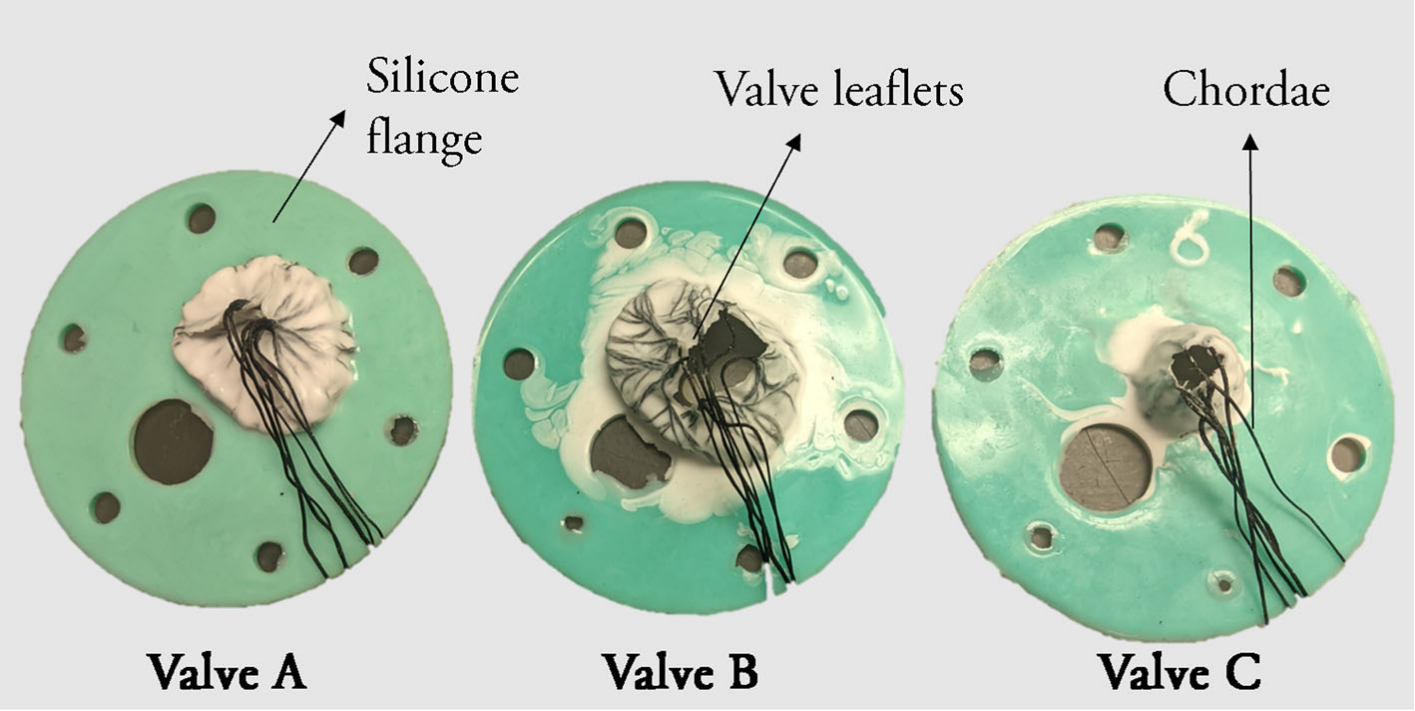
Three patient-specific tricuspid valves modeled using silicone and darcon strings

To achieve magnetic tracking (MT) of the ultrasound, we utilized the Aurora Tabletop Field Generator (NDI, Waterloo, Canada) and a 6-DoF sensor to track the ICE probe in 3D space during data collection.

LV Plus Simulator (Archetype Biomedical Inc., London, Canada) was used as a pulsatile heart phantom to simulate a ventricle and an atrial chamber. The phantom can be equipped with patient-specific valves, which includes the valve leaflets embedded in a silicone flange for support [34]. The details of the methods used in the modeling of TV are given in the next section. Three patient-specific valves are created using this technique.

### TV modeling procedure

The negative mold of a silicone flange (11 cm in diameter, 3 mm thick) with patient modeled tricuspid valves [34] was created using an Ultimaker S5 3D Printer (Ultimaker, Utrecht, Netherlands) and printed using ToughPLA filament. Approximately 3 cm of the ends of 30 cm of dacron string were frayed to mimic chordae tendineae. A 50:50 by weight mixture of previously degassed (– 0.8 atm at 1 min) Part A and Part B of Mold Star$^{\text {TM}}$ Eco Flex-003 was brushed on the TV valve mold leaflets. The Mold Star$^{\text {TM}}$ Eco Flex-003 was pigmented white with Silc-Pig Silicone Pigment to allow for easy visualization. The frayed ends of the dacron string were carefully placed onto the leaflets, with each leaflet being attached to two dacron strings. Once the dacron cordae tendinae were securely positioned, the leaflets were coated once more with the Mold Star$^{\text {TM}}$ Eco Flex-003 mixture. The silicone leaflets were allowed to cure for 30 min. To make the silicone flange surrounding the valve, a 50:50 by weight mixture of previously degassed (– 0.8 atm at 1 min) Part A and Part B of Mold Star$^{\text {TM}}$ Slow 15 was then poured into the mold. The silicone flange was allowed to cure at room temperature and pressure for 45 min prior to removal from the negative mold.

### Data collection

An MT sensor was attached externally to a Foresight™ ICE probe using an adhesive. Prior to imaging, the ICE probe (in Doppler mode) was spatially calibrated using a point-to-line registration method [35, 36].

The pulsatile heart phantom was placed over the table-top MT field generator (Fig. 2). The phantom was set to a normal rhythm at 60 beats per minute. Pure talc powder was used as an ultrasound contrast agent to enhance Doppler imaging. The ICE probe, in Doppler mode, was positioned in multiple locations at which a regurgitant jet could be observed. The regurgitation was produced via the patient-specific, pathological tricuspid valves fitted inside the beating heart phantom. Three TVs (valves A, B and C) were prepared and fitted consecutively to acquire data. It must be noted that valve C was a pediatric, infant valve which was comparatively smaller than valves A and B (Fig. 1).

Images were acquired from the Hummingbird console display using a frame-grabber (Epiphan, Ottawa, Canada) at a rate of 15 frames/second. The data were recorded using the Plus Server to communicate ultrasound and tracking information to 3D Slicer. For each of the three valves, five datasets were acquired, with each containing at least 5 seconds of imaging and tracking information. These data were processed to localize the vena contracta in 3D from the tracked, Doppler imaging of pathological tricuspid valves in real-time.

### Data processing

**Fig. 3:**
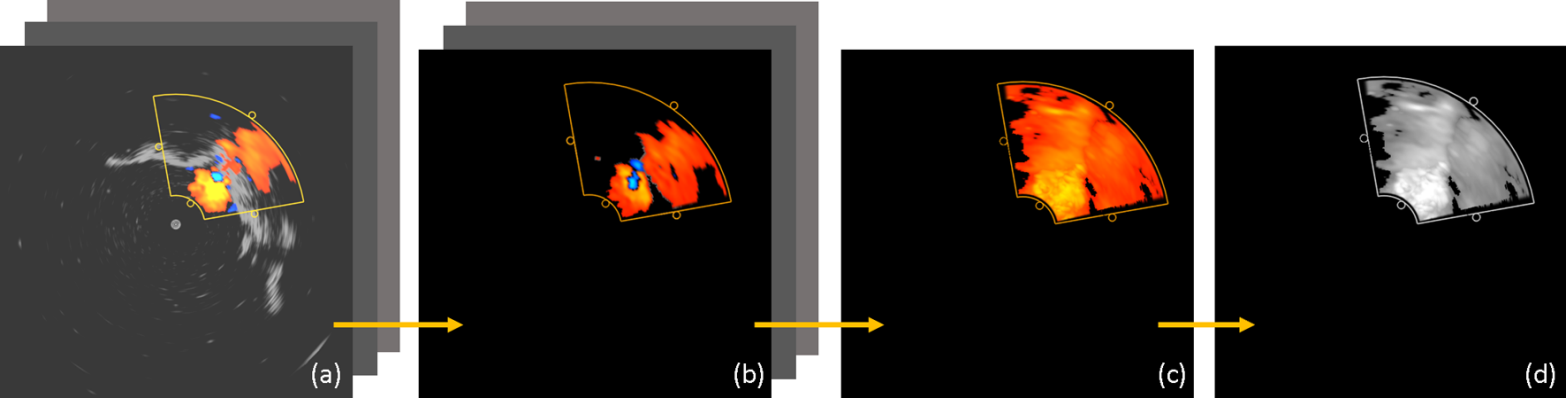
**a** Sequence of Doppler ICE imaging with maximum regurgitant flow. **b** Imaging sequence containing Doppler information only undergoes maximum intensity projection to create **c** a resultant image with all the highest-velocity Doppler information. This resultant image is then converted to **d** a grayscale image for further processing

The first step, to isolate the images with maximum regurgitation (Fig. 3a), was performed semi-automatically by the user. The peak valvular regurgitation usually occurs somewhere during the systolic phase of the cardiac cycle. To identify the exact phase of peak regurgitation, the user scans the first few images in a dataset to manually identify the first image exhibiting the highest regurgitation, along with the number of subsequent US images to be selected from each cardiac cycle. Then, all the images present at the selected cardiac phase were automatically isolated by our designed Slicer module using the data acquisition-rate information from the frame grabber and the beating rate selected of the heart phantom. These images were stored in a ‘Sequence’ in 3D Slicer.

This sequence of peak-regurgitant Doppler ultrasound images was then processed to remove all the grayscale, B-mode information from all the images. Since the objective is to isolate the vena contracta, the non-regurgitant blood flow (depicted in cool colors) was also removed from the images by suppressing the pixels with blue channel information. It must be noted that the quality of the regurgitant jet from individual cardiac cycles can sometimes be suboptimal. Therefore, to acquire an adequate jet image, all the images in the sequence were compounded together into one resultant image with the regurgitant flow (Fig. 3c). This step was achieved by using the maximum intensity projection (MIP) principle. In doing so, the most yellow pixel or the highest velocity information is retained in the resultant image.

**Fig. 4:**
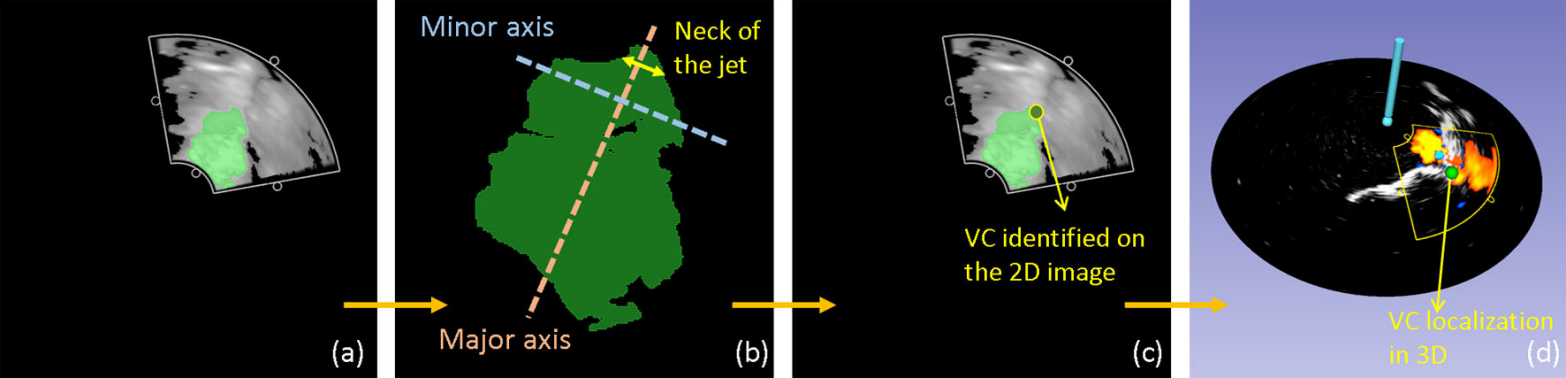
**a** Resultant Doppler image overlaid with the segmentation of the regurgitant jet. **b** Principal component analysis of the segmented region to derive the location of the vena contracta (VC). VC localization seen on **c** a 2D image and in **d** 3D tracking space

The resultant combined Doppler image contained the complete regurgitant jet, depicting blood flowing backward from the ventricle to the atrial chamber (Fig. 3c), and was converted to grayscale for further processing. The image was subjected to a binary threshold at an intensity of 150 to segment the brighter pixels representing the higher velocities in the regurgitant jet. For valve C, this threshold was set to 130 to accommodate the flow through a smaller, infant valve.

The next step was to identify the axis of the regurgitant jet. The segmented region was subjected to principal component analysis (PCA) to identify the major and minor axis of the jet, as well the principal moments. This information was used to transform the segmented region to lie along the major axis (Fig. 4b). The noise was removed by retaining only the largest connected island within the segmented region which was representative of the atrial regurgitant jet.

**Fig. 5:**
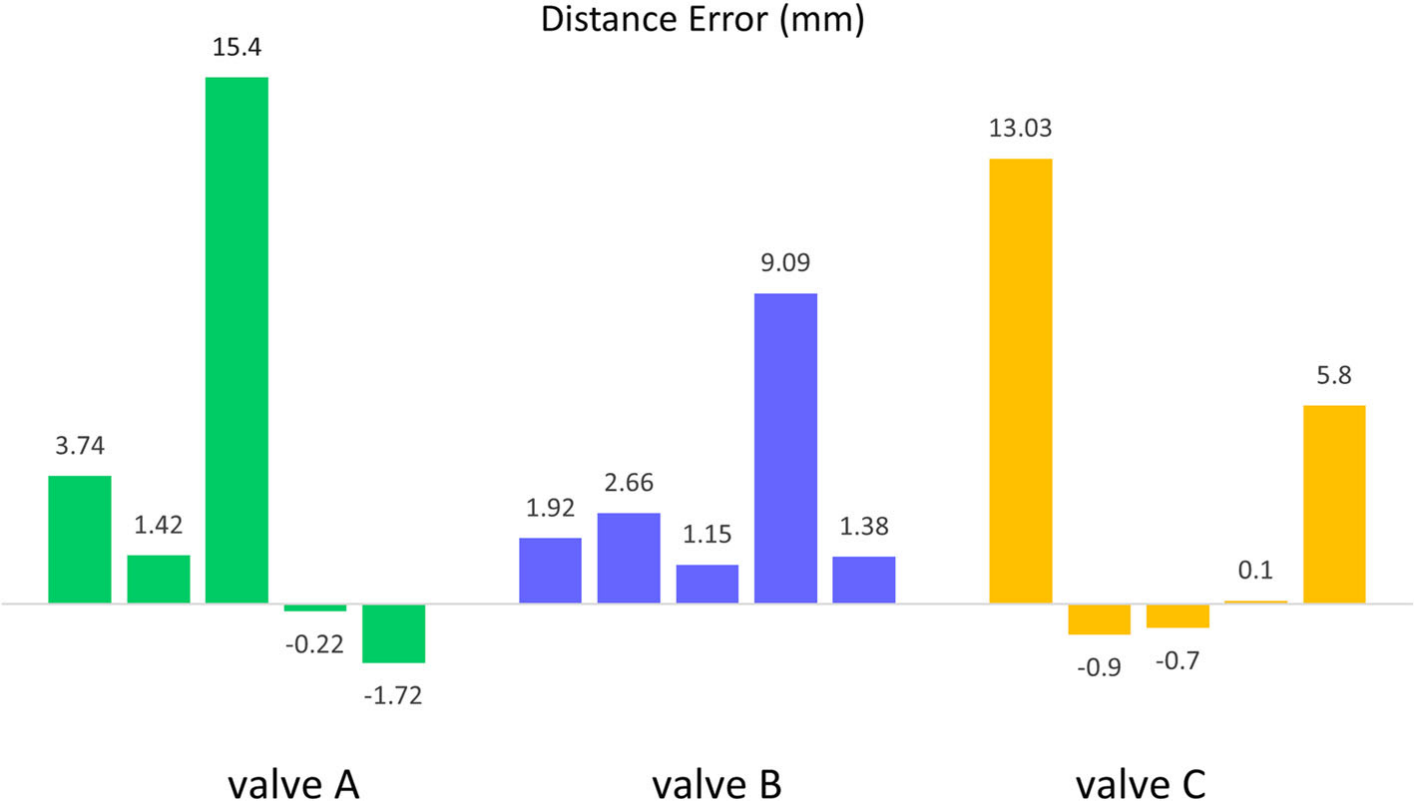
Error bars representing the minimum distance between the algorithm-detected vena contracta location and the ground truth model. For each of the valves, one high error bar can be seen as an outlier

**Fig. 6:**
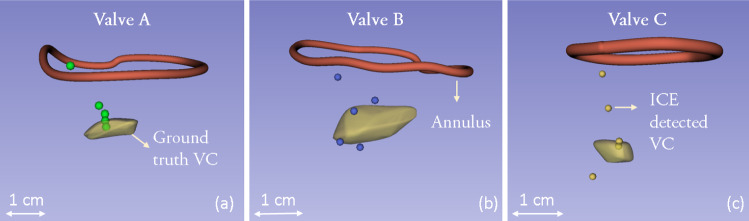
A qualitative analysis of the results showing the ICE-derived vena contracta locations as points and the ground truth vena contracta as a model (in yellow). A pre-mapped annulus model and vena contracta location in a tracked environment can provide more contextual landmarks for device positioning

From the transformed regurgitant jet, our proposed algorithm then identified the location of the vena contracta. At each point along the major axis, the height of the segmentation was measured, and the point with the minimum height was recorded. This minimum height was estimated as the vena contracta width (VCW), while the midpoint along the VCW was noted as the transformed vena contracta location. The inverse transformation from the PCA was applied to retrieve the original coordinates for the location of vena contracta in the US image. Figure 4 shows the VCW on the segmented jet region and the vena contracta location placed on a 2D ICE image.

The Foresight™ ICE images are conical in nature and lie in 3D space. The ICE image displayed on the console is a projection of the conical surface image along the height-axis. As such, the location of vena contracta on 2D images is not accurate and lacks the third dimension. Using the imaging angle information provided on the console screen, the location of the VC with respect to the true 3D image is calculated. The details of this conversion can be found in Nisar et al. [36]. Finally, the ICE probe calibration information and the probe location transform, provided by the EM tracking system, were applied to acquire the location of the VC in 3D space (Fig. 4d). This location represents the origin of the regurgitation in the tricuspid valve, which occurs most often at the coaptation gap.

### Validation

Prior to data collection for each valve, the ground truth VC and annulus were identified for validation. A pre-tracked and pre-calibrated needle was used to identify the VC in 3D tracking space, where the tip of the needle and the orientation of the needle shaft are tracked. The position of the ground truth of the VC was obtained by visually identifying and manually tracing the periphery of the regurgitant orifice using the tracked needle tip. The points were used to construct a 3D model of the ground truth vena contracta. Similarly, the outline of the annulus points was marked, and the model was constructed. The VC point locations detected by the algorithm were compared to the manually isolated ground truth VC model by estimating the closest distance between them.

## Results

For each of the valves, the distance between the ground truth VC model and the ICE-derived vena contracta locations were computed. The distance error for all the datasets can be seen in Fig. 5. As can be seen by the three tall peaks in the graph, there is one outlier case for each valve where the error is unacceptable. The outliers were a result of insufficient Doppler imaging as captured by the frame grabber. Across the three valves and excluding the three outliers, the average distance error between the detected VC and the ground truth model is 1.22 ±2 mm.

Qualitatively, the position of the ground truth vena contracta, corresponding to the coaptation gap, can be seen as an irregular shaped body in yellow in Fig. 6. The manually identified annulus ring is also represented to provide contextual information. The three high-error points can be seen near to the annulus in 3D which is a clear indication of these points as being incorrect and outliers. For valve A and B, the detected VC locations are close to the ground truth. The highest error was recorded in a dataset for valve C at 5.8 mm.

## Discussion

During interventions, clinicians rely on anatomical landmarks to guide and align the tools properly. Traditionally, the identification of landmarks and the positioning of tools takes place simultaneously, thus making the procedure intricate and demanding. Pre-mapping these landmarks can simplify these procedures by providing more information to the clinician while they position the devices. In this study, we present a method to semi-automatically extract the location of vena contracta, a clinically relevant landmark, from ultrasound images and represent it in 3D space. ICE imaging is used to generate 3D models of important anatomical features to potentially enhance the spatial awareness of the interventionalist as well as give information about the relative positioning of the procedural tools with the cardiac anatomy. Moreover, the presence of ICE allows the clinicians to acquire up-close live ultrasound imaging as the procedure is being performed. Since ICE probes can be manipulated to acquire non-traditional anatomical views, the technique presented in this paper can be particularly useful during complicated TV edge-to-edge repairs where the clip has to be positioned at more challenging positions such as at the posteroseptal and anterioposterior commissures [37].

The results from this study indicate that the designed 3D Slicer module can reliably localize the VC in most cases. Literature suggests that for cardiac interventions an error margin of up to 5 mm is acceptable [38]. In comparison, our average error of 1.22 ±2 mm is appropriate for this early-stage study. It should also be noted that the ground truth established in these experiments should be considered as a “bronze” standard as it was manually identified by visual characterization of the coaptation gap. Hence, it is susceptible to both human error and subjectivity.

A major limitation of this study is the presence of the outliers when the algorithm is unable to identify the VC accurately and instead the VC is localized near the annulus. In a use case, an outlier can be easily identified when the detected VC was positioned too close to the TV annulus. Outliers indicate that the valve should be reimaged and processed by the algorithm again. We suspect these outliers to be a result of the lower frame rate used in the study which meant that the regurgitation was not captured in the imaging data. During the experiments, it was observed that the recorded data in Slicer lacked some of the imaging frames showing high regurgitation on the Hummingbird console screen. The frame grabber was operating at a rate of 15 frames per second and in some cases missed capturing the image frame with the maximum regurgitation. To record the complete regurgitant Doppler imaging, we recommend using a frame grabber with a higher frame rate. Ideally, the imaging data should be transmitted directly from the ultrasound machine but this infrastructure is not yet available in most of the clinical console, including the Hummingbird console, used in this study.

A consideration while imaging the valve would be to use a narrower field of view for Doppler imaging to optimize and focus in the direction of the regurgitant jet. This simple factor can greatly enhance the overall efficiency of the designed algorithm.

Besides the VC, the annular ring of the tricuspid valve is another important landmark during TV interventions. In this study we manually identified the annulus ring; however, the procedures can benefit from automated ultrasound-based techniques to identify the TV annulus in 3D space. Future work can involve implementation of the existing methods in the literature that can extract and model the annulus from ultrasound. Li et al. [32] present a method for tracking the mitral valve annulus and it can potentially be adapted for TV annulus modeling as well.

Since the valves used in this study are modeled after real patient-specific TV, there is room for collecting more and complex tricuspid regurgitation cases. The valve modeling technique and the beating heart phantom allow mimicking realistic conditions, reducing the need for in vivo testing at such an early stage of the study. With a variety of TV models, the algorithm can be made more robust by testing and modifying it to accommodate more versatile patient cases. Future work can involve making the Slicer module more robust and suitable for even more complex tricuspid valve pathologies. The ultrasound guidance approach can also be enhanced with the emerging 4D ICE technology, like VeriSight Pro (Philips) and NuVision (Biosense Webster), which provides improved imaging of the subvalvular apparatus during transcatheter TV repair.

## Conclusion

Tricuspid valve interventions and related technology are evolving as more cases are being performed with imaging being a major challenge in them. A suitable alternative to the existing TEE-based workflows is to employ ICE imaging to visualize the anatomy. In this paper, we presented a method to provide more contextual information to the interventionalists during the TV repair procedures to reduce the regurgitation. A tracked ICE probe can be used to localize and pre-map significant landmarks in order to assist the meticulous task of device positioning during TV repair. Image guidance systems with mapping technology have successfully simplified complex cardiac procedures like ablation therapy [39]. This study is one step toward using image guidance for tricuspid valve interventions to potentially streamline the challenging TV repair procedures.

## Data Availability

The 3D Slicer module, test data and a video demonstration can be found at https://github.com/hareem-nisar/VC-localization.

## Code availability

Complete code is available at https://github.com/hareem-nisar/VC-localization.
